# Prenatal Magnetic Resonance Imaging Assessment of Fetal Airway Dimensions: Establishing an Anthropometric Database

**DOI:** 10.1002/pd.70140

**Published:** 2026-04-02

**Authors:** Juliette Houssin, Ahmed El Ahmadi, Robinson Gravier‐Dumonceau, Richard Nicollas, Eric Moreddu

**Affiliations:** ^1^ Pediatric Otorhinolaryngology‐Head & Neck Surgery La Timone Children's Hospital APHM Aix‐Marseille Univ Marseille France; ^2^ Imaging Department Hopital Nord APHM Aix Marseille Univ Marseille France; ^3^ BioSTIC Department of Biostatistics and Information and Communication Technologies Assistance Publique‐Hopitaux de Marseille (AP‐HM) La Timone Hospital Marseille France

## Abstract

**Objective:**

Fetal airway anomalies can severely impair breathing at birth, potentially causing brain injury or death. Thus, early prenatal diagnosis is essential. While MRI is the most effective imaging modality for evaluating fetal airways, normative MRI data remain scarce.

**Method:**

This retrospective study analyzed fetal MRIs performed at a tertiary‐care center between January 2, 2018, and October 11, 2023. Measurements of the trachea and pharynx—including diameters, lengths, and volumes—were collected from axial, sagittal, and coronal slices. Statistical tests included ANOVA, *t*‐test, Wilcoxon, chi‐square, and Fisher's exact tests, with significance set at *p* ≤ 0.05.

**Results:**

MRI data from 278 fetuses—either healthy or with non‐airway‐affecting anomalies—were analyzed. Airway dimensions significantly increased with gestational age. Tracheal axial diameter ranged from 1.70 to 2.00 mm (*p* = 0.001), coronal diameter from 2.20 to 3.10 mm (*p* = 0.001), and sagittal length from 33.1 to 35.9 mm (*p* = 0.003) between the fifth–sixth and ninth gestational months. Head position influenced pharyngeal but not tracheal measurements.

**Conclusion:**

This study provides the first MRI‐based normative data for fetal airways, offering valuable reference values for prenatal assessment. MRI proves effective in monitoring airway development and planning neonatal respiratory management.

## Summary

- What's already known about this topic?
  ◦ Airway pathologies can be life‐threatening for the fetus right from birth.
  ◦ To date, fetal upper airway anomalies have only been described qualitatively in small cohorts.
- What does this study add?
  ◦ Measurement references including tracheal and pharyngeal diameters and lengths across all slices
  ◦ Describe influence of head position on pharyngeal, but not tracheal, dimensions.
  ◦ Providing an anthropometric database that can serve as a reference for clinicians and researchers in prenatal diagnostics and fetal medicine.

## 1 Introduction

Among fetal congenital anomalies, pathologies affecting the airway patency can result in respiratory depression at birth, which can have irreversible neurological consequences, even leading to death [1, 2]. Therefore, the anatomical description and monitoring of potential fetal airway abnormalities are essential in prenatal diagnosis, parental counseling, and planning possible medical‐surgical management at birth.

In recent years, fetal MRI has increasingly been used as a diagnostic tool in prenatal diagnostics [3], providing high‐contrast images of soft tissues and fluid‐filled structures during fetal life [4]. MRI allows for better characterization and monitoring of tissular masses, structural defects, and developmental anomalies within congenital syndromes compared with ultrasound [4, 5, 6, 7, 8]. The quality of MRI images is less affected by fetal position, potential ossifications, oligohydramnios, or maternal obesity [9]. Regarding airways, MRI can reveal tracheal deviations and improve the diagnosis of airway obstructions [1, 2, 10, 11]. It is performed from 20 gestational weeks (GA), at which point organ visualization becomes interpretable [6]. The main goal of prenatal diagnosis is to determine the fetus' postnatal viability and, if necessary, plan management at birth to address predictable ventilation complications. An EXIT (ex utero intrapartum therapy) or a PRESTO (Procedure Requiring Second Team in the OR) may be considered for childbirth, allowing the fetal airways to be secured. During an EXIT, oxygenation is maintained via placental circulation. This intervention anticipates extreme emergencies but carries significant risks for both the mother and fetus, requiring precise indications [5, 7, 10, 12, 13].

Due to the rarity of congenital airway pathologies, published studies in the literature include only limited cohorts, lacking the power to identify factors that could guide prenatal diagnosis more precisely than indirect signs. Airway descriptions remain qualitative [4, 8]. A more detailed description of airway morphology and its evolution based on gestational age is needed, conducted on a large cohort to distinguish more precisely pathological from normal and monitor diagnosed anomalies. Dave et al. reviewed existing literature on airway dimensions from fetal to adolescent age, revealing great heterogeneity in results. Moreover, studies reporting MRI measurements included only children or newborns [14].

Due to the disparity in measurements based on the interpretation method used and the lack of reference standards for analyzing airways via MRI, this study aims to present an anthropometric baseline of airway dimensions as described by fetal MRI.

## 2 Materials and Methods

### 2.1 Patient Inclusion

Fetal MRIs performed at the Radiology Department of our University Hospital, from January 2018 to October 2023, were retrospectively reviewed, corresponding to a period of 5 years and 9 months. All images were obtained using the Siemens Magnetom 1.5 T MRI (Siemens Medical Systems, Erlangen, Germany). The 1.5 T MRI was more suitable than the 3 T MRI as the images were less artifacted by fetal movements. The sequences used for airway measurements were T2 TrueFISP sequences. Indications included the exploration of suspected or diagnosed thoracic, abdominal, or spinal pathologies during pregnancy follow‐up ultrasounds. Exclusion criteria were the absence of all available slices, poor image quality due to excessive fetal or maternal movement, poor‐quality examination, twin pregnancies, severe malformations rendering airways uninterpretable, and fetuses with conditions that could affect the airway dimensions.

### 2.2 Collected Data

Due to the limitations of fetal MRI in identifying certain structures, simplifications were made in naming different parts of the airways. The trachea (including the trachea and larynx), the pharynx (extending from the nasopharynx to the hypopharynx), and the carina were distinguished. Images were analyzed in axial, sagittal, and coronal slices. The technique used to measure the trachea and pharynx, in terms of diameter and length, was chosen to be as reproducible as possible for any practitioner confronted with the interpretation of the airways on fetal MRI.

**FIGURE 1:**
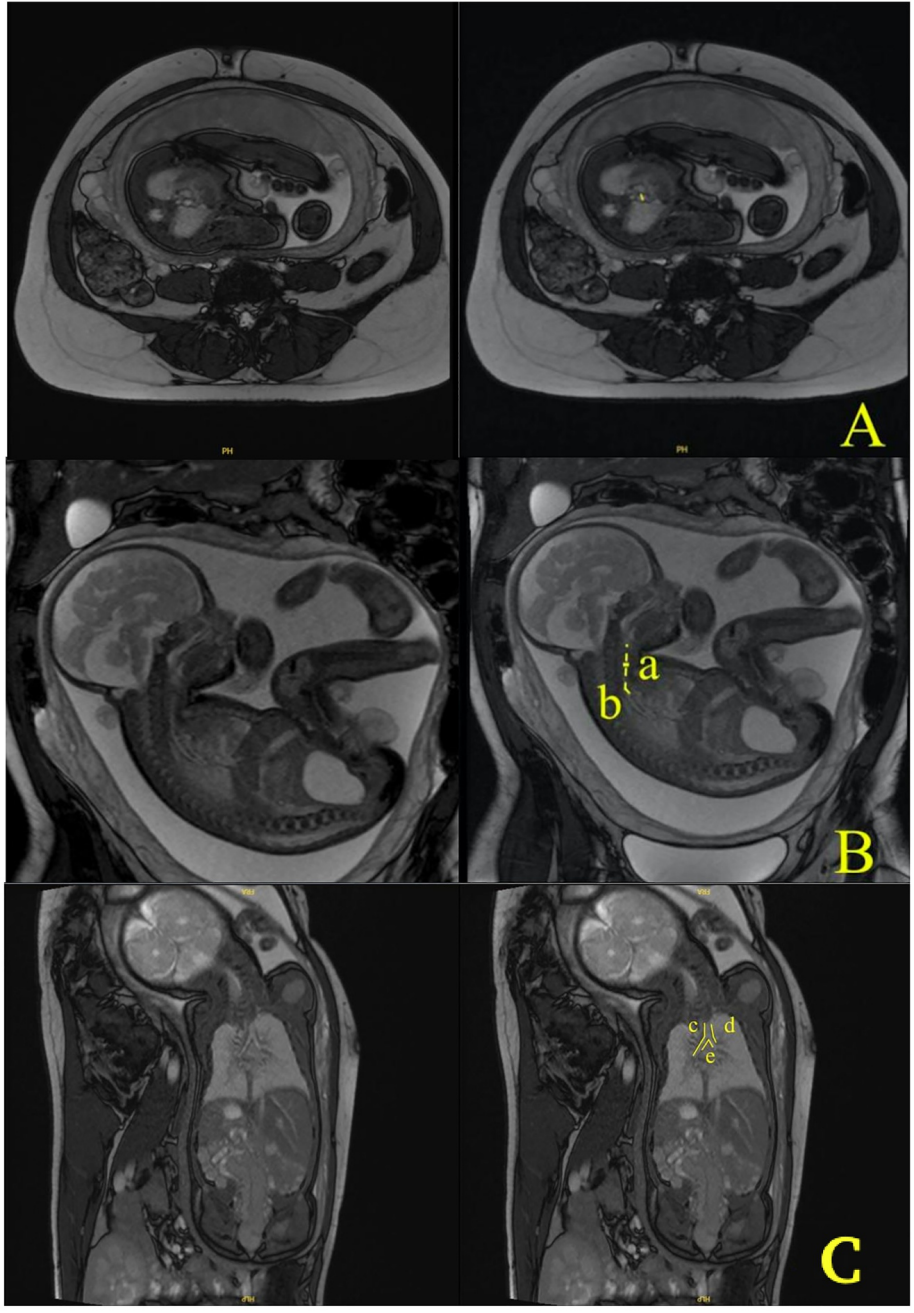
Measurement of the tracheal diameter on axial slice (A) of the diameter (a) and length (b) of the trachea on sagittal slice (B), and of the left angle (c), right angle (d), and carinal angle (e) of the trachea on coronal slice (C).

On axial slices, measurements included:

- Maximum and minimum tracheal diameter (Figure 1A);
- Pharyngeal surface area (on the slice with the maximum surface area);
  On sagittal slices, measurements included:
- Total measurable tracheal length;
- Tracheal diameter measured at mid‐height of the trachea (Figure 1B);
- Pharyngeal area (measured from the nasopharynx to the hypopharynx) on the slice with the maximum surface area;
- Degree of head flexion analyzed by the angle measured between the nasal floor and a line parallel to the sagittal axis of the thorax.

On coronal slices, measurements included:

- Tracheal diameter (measured above the carina);
- Subcarinal angle;
- Angle between the upper wall of the left main bronchus and the left lateral wall of the trachea;
- Angle between the upper wall of the right main bronchus and the right lateral wall of the trachea (Figure 1C);
- Degree of lateral head inclination measured by the angle between the head axis identified, if possible, using the cerebral falx and the coronal axis of the thorax;
  For statistical reasons, measurements of carinal angles were grouped into four categories for the subcarinal angle and five categories for the left and right angles. The distribution of angle measurements by group was analyzed without statistical testing by comparing percentage distributions for each group.

To assess the relative position of the fetus's head to the thorax, the fetal presentation was noted.

All measurements were performed by the same examiner. A second series of all the measurements was conducted for 27 fetuses by the same examiner to calculate intra‐examiner variability, and a series of measurements was conducted for 27 fetuses by another examiner to calculate inter‐examiner variability. Both series were compared with the first series using an ANOVA‐test (*p* < 0.05).

### 2.3 Statistical Analysis

Measured MRI values were compared based on sex, presence of hydramnios, fetal presentation, sagittal head inclination, and lateral head inclination. Note that for the comparison by fetal presentation, the “Deflexed head” group was excluded from statistical analysis as it contained only one subject. Ages were grouped by month (fifth month for 20 and 24 GA, sixth month for 25–28 GA, seventh month for 29–32 GA, eighth month for 33–36 GA, and ninth month for 37–41 GA). For comparison by pregnancy month, observations for the fifth and sixth months were grouped due to sample size (*n* = 1 in the fifth month). Continuous variables were described by their mean and standard deviation. When comparing the two groups, the Student t‐test or Wilcoxon test were used depending on the conditions. When comparing more than two groups, an ANOVA or a Kruskal‐Wallis test were used depending on the conditions. Categorical variables were described by their count and percentage and compared using the chi‐square test or Fisher test depending on the conditions. Two‐tailed tests were conducted and considered statistically significant for *p* ≤ 0.05. Statistical analysis was performed using R software (version 4.2.0).

## 3 Results

**FIGURE 2:**
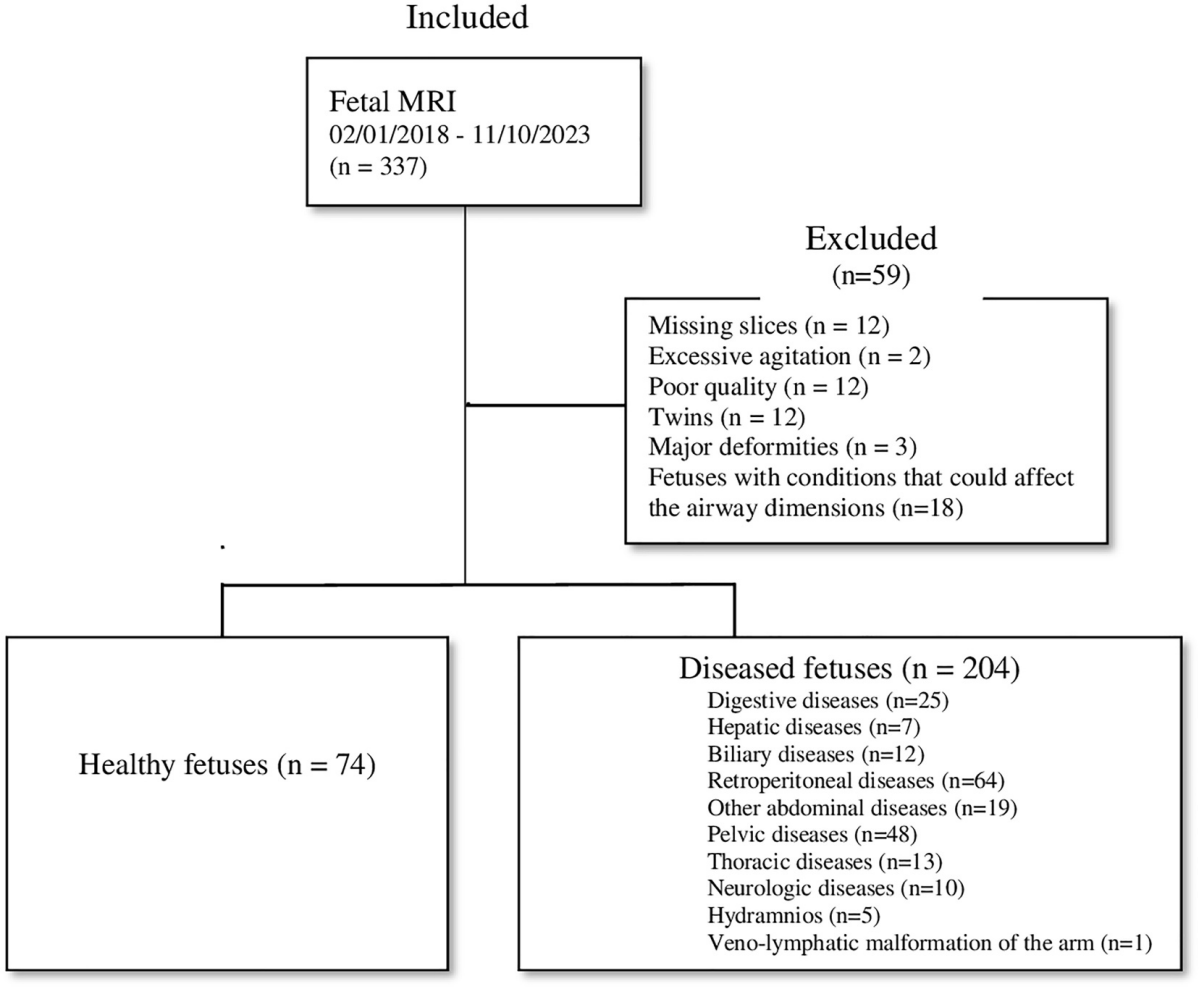
Flow‐chart of the studied population.

**FIGURE 3:**
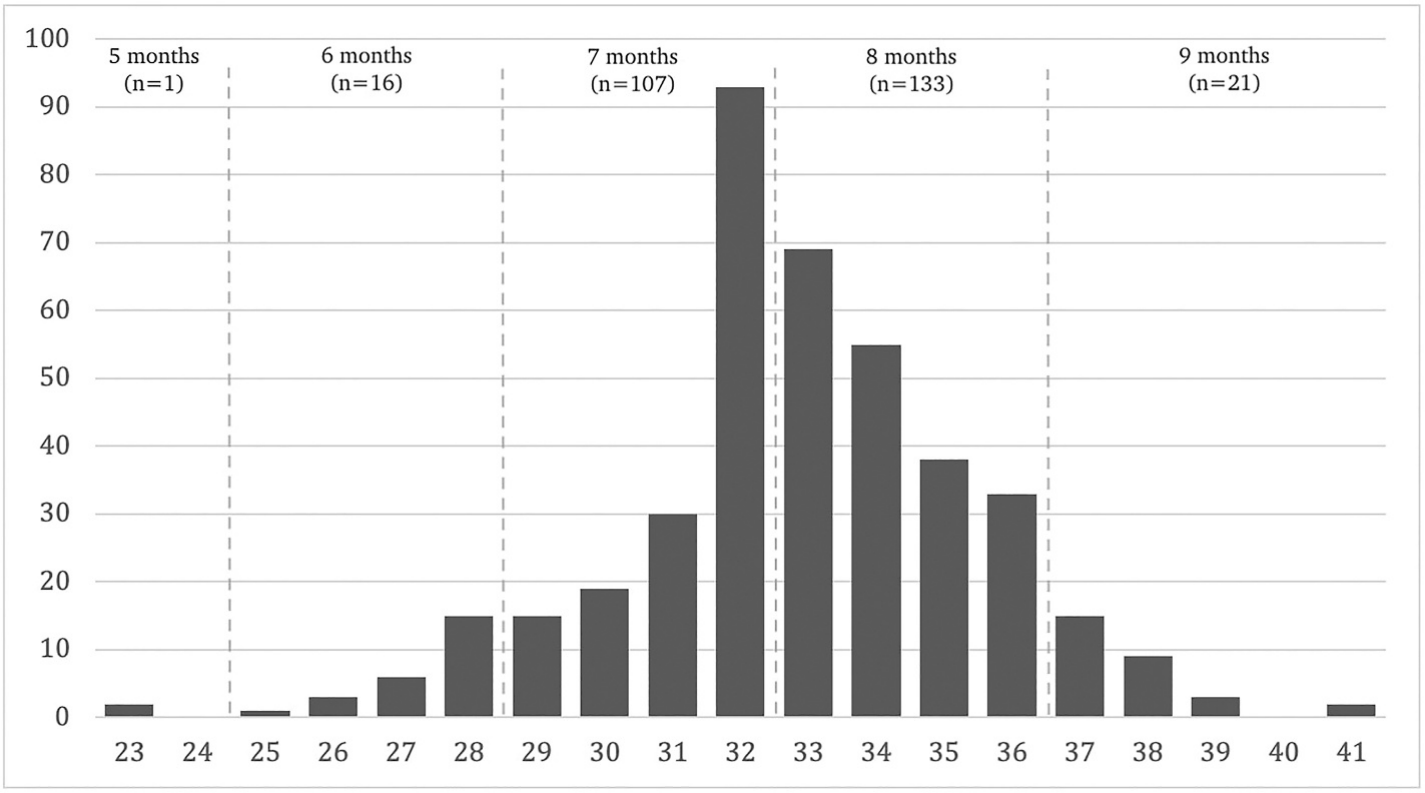
Distribution of the population by gestational age in weeks of amenorrhea.

MRI images of exams performed over almost 6 years, from January 2, 2018, date of first use of the Siemens Magnetom 1.5 T MRI (Siemens Medical Systems, Erlangen, Germany), to October 11, 2023, end date of study, were reviewed. 278 fetuses were included: 74 fetuses without abnormalities, and 204 with abnormalities not affecting the airways (Figure 2). The fetuses included were aged 23–41 GA (Figure 3).

**FIGURE 4:**
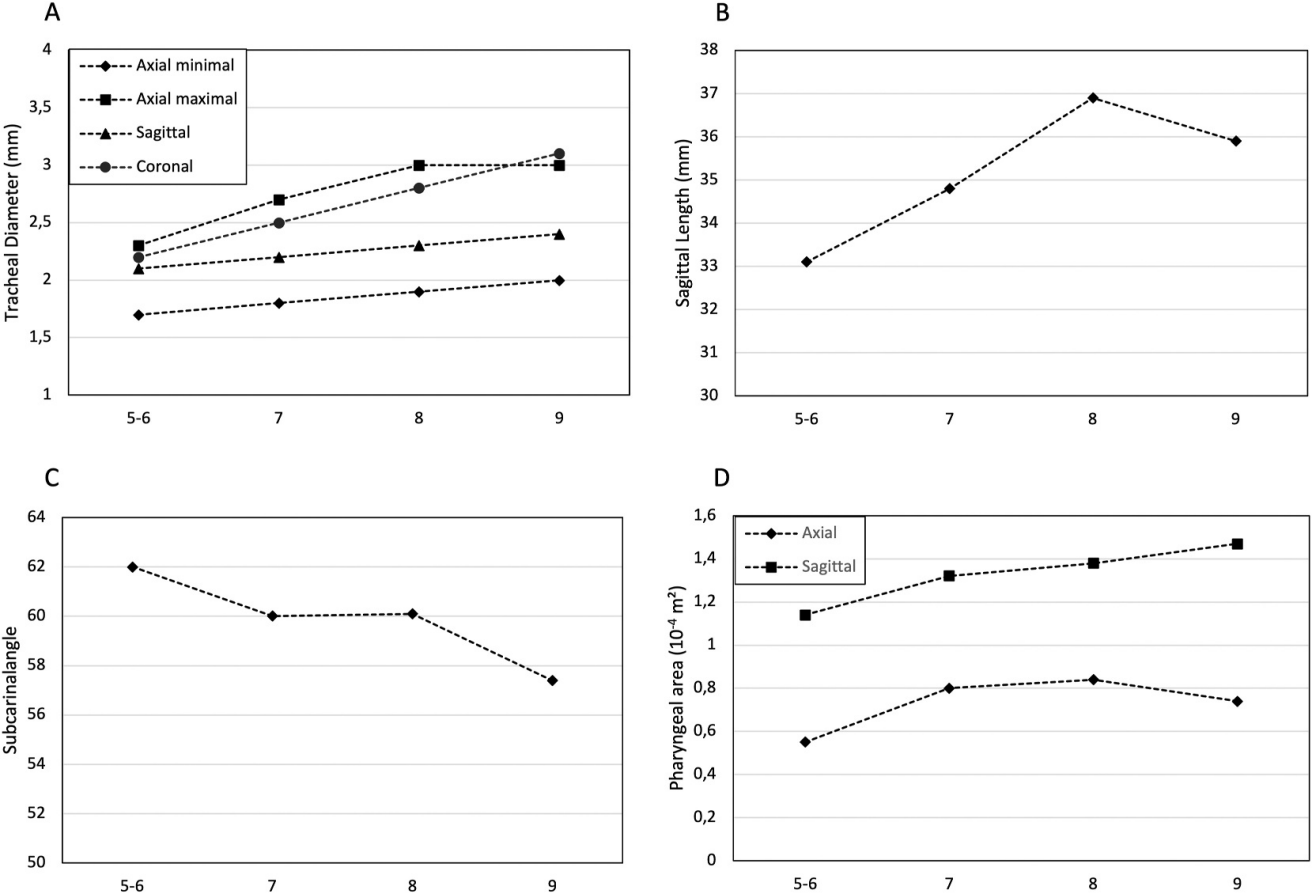
Minimal axial and maximal axial diameter, sagittal and coronal diameter (A) in 10^−3^ m, sagittal length (B) in 10^−3^ m, carinal angles (C) in degrees, and axial and sagittal pharyngeal area (D) in 10^‐^4 m^2^ by pregnancy month. In C, angles represent the mean for each category for all the group of fetuses.

The dimensions of the fetal airways measured by MRI according to pregnancy month are presented in Table 1 and Figure 4. Tracheal diameter and length, as well as pharyngeal area, values were also described by weeks of amenorrhea (Supporting Information S1: Appendice 1). Values significantly increased with pregnancy month concerning the minimal and maximal tracheal diameters measured axially (Figure 4A), sagittal diameter and length of the trachea (Figure 4A and B), coronal diameter of the trachea (Figure 4A), and axial pharyngeal area (Figure 4D) (Table 1). There was no statistically significant difference by pregnancy month concerning the sagittal pharyngeal area (Figure 4D) and carinal angles (Figure 4C).

**TABLE 1:** Measured values by MRI by pregnancy month (median (interquartile range)).

Measurement	5–6 Month (*n* = 17)	7 Month (*n* = 107)	8 Month (*n* = 133)	9 Month (*n* = 21)	*p* value
Trachea					
Axial					
Minimal diameter	1.70 (1.50–1.80)	1.80 (1.60–2.10)	1.90 (1.70–2.30)	2.00 (1.80–2.30)	0.002*
Maximal diameter	2.30 (2.20–2.70)	2.70 (2.40–3.05)	3.00 (2.50–3.40)	3.00 (2.70–3.60)	< 0.001*
Sagittal					
Diameter	2.10 (1.90–2.20)	2.20 (2.00–2.60)	2.30 (2.00–2.60)	2.40 (2.10–2.90)	0.065
Length	33.1 (24.4–33.7)	34.8 (28.2–41.6)	36.9 (31.9–43.0)	35.9 (33.4–45.4)	0.003*
Coronal					
Diameter	2.20 (1.70–2.40)	2.50 (2.20–3.00)	2.80 (2.50–3.30)	3.10 (2.70–3.60)	< 0.001*
Subcarinal angle	62.0 (54.0–68.8)	60.0 (50.0–71.0)	60.1 (48.2–68)	57.4 (48.0–66.8)	
Left angle	148 (131–157)	140 (132–149)	143 (133–151)	140 (124–154)	
Right angle	152 (142–159)	161 (146–169)	158 (147–164)	152 (145–170)	
Other measurements					
Axial pharyngeal area	0.55 (0.49–0.68)	0.80 (0.55–1.01)	0.84 (0.60–1.16)	0.74 (0.53–0.93)	0.012*
Sagittal pharyngeal area	1.14 (0.76–1.39)	1.32 (1.01–1.67)	1.38 (1.12–1.79)	1.47 (1.09–2.08)	0.068

*Note:* Tracheal diameters and lengths are expressed in mm, area in 10^−4^ m^2^, volume in 10^−6^ m^3^ and angles in degrees. An asterisk indicates significant results.

### 3.1 Comparative Analysis

There was a statistically significant difference by gender for sagittal tracheal diameter, 2.23 (± 0.48) mm for female fetuses versus 2.37 (± 0.50) mm for male fetuses (*p* = 0.017). No statistically significant difference was found for the other evaluated values (Supporting Information S1: Appendice 2).

There was no statistically significant difference found by fetal presentation for all the measured values (Supporting Information S1: Appendices 3 and 4).

There was a statistically significant difference between the groups by sagittal head inclination. The sagittal tracheal length was statistically different for the “hyperextension” group (44.9 mm), “head flexed” group (35.8 mm) and “in axis” group (35.9 mm) (*p* = 0.034). The axial pharyngeal area was statistically different with 0.91 cm^2^ for the “head flexed” group, 0.73 cm^2^ for the “in axis” group and 0.89 cm^2^ for the “hyperextension” group (*p* = 0.018), and also was the sagittal pharyngeal area, with 1.36 cm^2^ in the “head flexed” group, 1.54 cm^2^ in the “in axis” group and 1.66 cm^2^ in the “hyperextension” group (*p* = 0.031). No statistically significant difference was found for other measured values (Supporting Information S1: Appendices 5 and 6).

For lateral head inclination, a difference was observed only for the right bronchial angle measurement, with a majority of right bronchial angle values between 140 and 160° for the “head tilt” group versus a majority of values between 160 and 180° for the “on axis” group (*p* = 0.04). No other values varied significantly (Supporting Information S1: Appendices 7 and 8).

The ANOVA test comparing the measures repeated by the same examiner and the other examiner did not reveal any significant intra‐examiner and extra‐examiner variability.

## 4 Discussion

### 4.1 Principal Findings

This study is the first to present tracheal and pharyngeal dimension norms as described by MRI from a large cohort (*n* = 278). Significant increases in tracheal and pharyngeal dimensions were observed with gestational age. Comparative analysis found a difference by sex for sagittal tracheal diameter and no statistically significant differences for all the other measurements. There was a statistically significant difference concerning tracheal length and axial and sagittal pharyngeal area according to sagittal head inclination.

### 4.2 Results in the Context of What Is Known

Available literature only qualitatively describes the trachea as it appears on MRI, noting morphological anomalies such as the presence of intraluminal soft tissue deviation or focal caliber reduction [4]. Indirect signs of airway obstruction, such as hydramnios secondary to impaired swallowing, are also sought [15, 16]. However, diagnosing conditions such as tracheal agenesis via MRI remains challenging, as it can go unnoticed if only hydramnios of unknown etiology is present [17]. Aoki et al. described parametric indices for identifying tracheal agenesis, tested on 8 fetuses with airway obstructions [18]. Lazar et al. developed a Tracheoesophageal Displacement Index (TEDI) correlated with complicated neonatal ventilation at birth if greater than 12, suggesting the need for an EXIT procedure [19]. Ng et al. identified the largest vertical dimension of the amniotic sac and mass effect on the trachea as independent predictors of significant morbidity in fetuses with cervicofacial masses [20]. Cash et al. found that the best indicators for airway intervention at birth were hydramnios, compression of cervical vessels, neck hyperextension due to mass, and severe micrognathia, based on a cohort of 27 fetuses [12]. Among these articles, several authors have criticized the lack of reference data on airway dimension norms from such a cohort as a comparison element for their work [18, 21].

#### 4.2.1 Effect of Fetal Sex

Analysis by sex found a statistically significant difference only for sagittal tracheal diameter (*p* = 0.017). Given the dimensional difference of about a tenth of a millimeter, the relevance of this result must be discussed. It seems legitimate to assume that sagittal tracheal diameter, like other observed values, is reasonably reproducible for both female and male sexes.

#### 4.2.2 Effect of Fetal Presentation and Head Inclination

We hypothesized that fetal presentation, particularly the head's relative position to the thorax, could influence the measured tracheal and pharyngeal dimensions. Fetal presentation seems to have a minor impact on the evaluated values. However, the analysis showed that parallel to head hyperextension, the measured sagittal tracheal length increased. Anatomically, this result can be explained by increased tracheal traction from cephalic hyperextension, increasing its measurable length. The trachea is designed to lengthen and shorten according to the position of the head; hence, its ring structure is interrupted by connective tissue. It should be noted that the upper limit of the trachea is identified on MRI by the marked narrowing observed below the pharynx, as the cricoid is not precisely identifiable. Some measured tracheal surfaces of high‐positioned slices correspond more precisely to the hypopharynx and larynx than to the trachea itself. The effect of head hypertension on the measured maximum tracheal surface area echoes that found for pharyngeal dimensions. Indeed, axial and sagittal measured pharyngeal areas differed significantly by head flexion or hyperextension, explained by the pharynx's pivotal position in the neck. Finally, lateral head inclination analysis showed a difference only for the right bronchial angle measurement. According to the analysis of group size in different angle measurement groups, the right bronchial angle is smaller when the head is aligned compared with fetuses with highly laterally inclined heads. Thus, lateral head inclination would affect angle measurements. However, the populations of “aligned head” and “tilted head” fetuses are similar in size. Therefore, it should be acknowledged that this isolated difference does not significantly affect the results presented in this study. Measurements of subcarinal angles are of interest in the search for the impact of anomalies on the airways. A narrowed carinal angle and, by extension, increased subcarinal angles are observed in diaphragmatic hernias. These angles may also be altered in cases of severe pulmonary sequestration and situs inversus.

#### 4.2.3 Comparison With Published Literature Values

Airways have been previously described as they can be analyzed on ultrasound by Kalache et al., finding a mean fetal tracheal diameter at 2.63 (± 0.20) mm at 25 GA [22], of a cohort of 198 fetuses. Szpinda et al. published tracheal measurements from 73 fetuses obtained after anatomical pathology examination, with a mean tracheal length measuring 20.77 (± 0.50) mm at 25 GA, and a mean external transverse diameter measuring around 5 mm at 25 GA [21, 23].

Several studies have examined fetal airway dimensions and their evolution based on gestational age, using an anatomopathological examination of post‐mortem fetuses. Daroszewski et al. measured the tracheobronchial angles of 73 fetuses, finding them independent of sex and age [24]. Harjeet et al. measured tracheobronchial dimensions of 40 fetuses, revealing linear growth of tracheal diameter without significant angle increase and no sexual dimorphism [22]. They also measured laryngeal, epiglottis, and cricoid cartilage dimensions, finding a positive correlation between various measurements and cranio‐caudal length [25]. Fayoux et al. conducted an anatomical study of thyroid and cricoid cartilage in 274 fetuses to describe laryngotracheal structural development, aligning with similar work by Cicekcibasi et al. [26, 27].

A first comparison of anatomical dimensions with imaging data was conducted by Miklaszewska et al., finding that ultrasound‐measured laryngeal dimensions exceeded anatomical dimensions from post‐mortem examinations [28].

Due to the sample size disparity and to obtain more reliable values from larger samples, age was categorized by month rather than by weeks of amenorrhea in this study, unlike previously published studies. However, to make the data comparable with other studies, an annexed table presents the obtained values for age categorization by weeks of amenorrhea. This table shows that ultrasound‐measured values are higher than those described by MRI, consistent with Miklaszewska et al.'s work [29]. Aoki et al. reported a median sagittal diameter measured by MRI of 3.1 mm in 30 control fetuses with a median age of 32 GA [15], compared to a median sagittal diameter of 2.3 mm found in the 32 GA fetus group (*n* = 63) in our study. Anatomical pathology data published by Gathe et al. from a study of 40 fetuses aged 12–40 GA reveal tracheal measurements higher than those obtained in this study [30]. This underscores the importance of presenting measurements specifically taken on MRI to make them comparable to fetal MRI conducted for prenatal diagnostic purposes.

### 4.3 Clinical Implications

To date, the assessments of dilation or narrowing described in the literature are always qualitative and evaluated based on the examiner's experience. Among the values noted in this large cohort, the minimal axial diameter could serve as a reference in cases of airway obstruction suspicion. Conversely, the maximal axial diameter could help detect cases of tracheal dilation. Pharyngeal measurements are intended to serve as a reference for identifying cases of pharyngeal dilation observed in CHAOS (Congenital High Airway Obstruction Syndrome).

### 4.4 Research Implications

This study aims to serve as a benchmark for evaluating images of fetuses' airways, using MRI. In addition, the data published in this study are intended to serve as a comparison element for future studies on fetal upper airway pathologies.

### 4.5 Strengths and Limitations

#### 4.5.1 Technical Comments

The chosen MRI was the 1.5 T, despite the recent development of the 3 T. This choice was made because the cohort that underwent imaging using the 1.5 T MRI was larger than that using the 3‐T MRI, and the 3‐T MRI does not offer a benefit over the 1.5 T MRI in airway description, as reported by Victoria in a retrospective study of 58 fetuses. They also discuss the development of 3D‐3T‐SSFP sequences for volumetric reconstructions and even virtual bronchoscopies to improve airway obstruction diagnosis [31].

## 5 Conclusion

This study presents an anthropometric baseline of airways as observed by MRI, which is now a frontline diagnostic tool in prenatal diagnosis. After presenting the found values concerning various measurable airway dimensions, several factors that could influence these values were tested, finding an effect of head position on observable pharyngeal dimensions without affecting tracheal dimensions. These values are intended to serve as a guide for assessing the airways of fetuses suspected of having upper airway pathologies.

## Funding

The authors have nothing to report.

## Ethics Statement

The use of the center's data has been approved by the ethics committee of Assistance Publique‐Hopitaux de Marseille, DRS (Direction de la Recherche Santé).

## Consent

The authors have nothing to report.

## Conflicts of Interest

The authors declare no conflicts of interest.

## Supporting information


Supporting Information S1

## Data Availability

The data that support the findings of this study are available from the corresponding author upon reasonable request.

## References

[1] W. Zheng , S. Gai , J. Qin , F. Qiu , B. Li , and Y. Zou , “Role of Prenatal Imaging in the Diagnosis and Management of Fetal Facio‐cervical Masses,” Scientific Reports 11, no. 1 (2021): 1385, 10.1038/s41598-021-80976-4. 33446872 PMC7809128

[2] G. Ryan , S. Somme , and T. M. Crombleholme , “Airway Compromise in the Fetus and Neonate: Prenatal Assessment and Perinatal Management,” Seminars in Fetal and Neonatal Medicine 21, no. 4 (2016): 230–239, 10.1016/j.siny.2016.03.002. 27084444

[3] L. Manganaro , A. Antonelli , S. Bernardo , et al., “Highlights on MRI of the Fetal Body,” Radiologia Medica, La 123, no. 4 (2018): 271–285, 10.1007/s11547-017-0834-7. 29164364

[4] E. I. Rubio , “Imaging of the Fetal Oral Cavity, Airway and Neck,” Pediatric Radiology 51, no. 7 (2021): 1122–1133, 10.1007/s00247-020-04851-6. 33978788

[5] A. Ravelli , M. Napolitano , M. Rustico , et al., “Prenatal MRI of Neck Masses With Special Focus on the Evaluation of Foetal Airway,” Radiologia Medica, La 124, no. 9 (2019): 917–925, 10.1007/s11547-019-01049-1. 31175537

[6] A. C. Coblentz , S. R. Teixeira , D. M. Mirsky , A. M. Johnson , T. Feygin , and T. Victoria , “How to Read a Fetal Magnetic Resonance Image 101,” Pediatric Radiology 50, no. 13 (2020): 1810–1829, 10.1007/s00247-020-04768-0. 33252751

[7] M. K. Dighe , S. E. Peterson , T. J. Dubinsky , J. Perkins , and E. Cheng , “EXIT Procedure: Technique and Indications With Prenatal Imaging Parameters for Assessment of Airway Patency,” RadioGraphics 31, no. 2 (2011): 511–526, 10.1148/rg.312105108. 21415194

[8] H. Shinmoto , K. Kashima , Y. Yuasa , et al., “MR Imaging of Non‐CNS Fetal Abnormalities: A Pictorial Essay,” RadioGraphics 20, no. 5 (2000): 1227–1243, 10.1148/radiographics.20.5.g00se071227. 10992014

[9] T. Wataganara , A. Ebrashy , L. D. Aliyu , et al., “Fetal Magnetic Resonance Imaging and Ultrasound,” Journal of Perinatal Medicine 44, no. 5 (2016): 533–542, 10.1515/jpm-2015-0226. 27092644

[10] G. Tonni , R. Granese , E. F. Martins Santana , et al., “Prenatally Diagnosed Fetal Tumors of the Head and Neck: A Systematic Review With Antenatal and Postnatal Outcomes Over the Past 20 Years,” Journal of Perinatal Medicine 45, no. 2 (2017): 149–165, 10.1515/jpm-2016-0074. 27508950

[11] B. Ulm , D. Muin , A. Scharrer , D. Prayer , G. Dovjak , and G. Kasprian , “Prenatal Ultrasound and Magnetic Resonance Evaluation and Fetal Outcome in High‐Risk Fetal Tumors: A Retrospective Single‐Center Cohort Study Over 20 Years,” Acta Obstetricia et Gynecologica Scandinavica 99, no. 11 (2020): 1534–1545, 10.1111/aogs.13933. 32525215 PMC7689914

[12] H. Cash , R. Bly , V. Masco , et al., “Prenatal Imaging Findings Predict Obstructive Fetal Airways Requiring EXIT,” Laryngoscope 131, no. 4 (2021): E1357–E1362, 10.1002/lary.28959. 32770766

[13] A. Spiers , G. Legendre , F. Biquard , P. Descamps , and R. Corroenne , “Ex Utero Intrapartum Technique (EXIT): Indications, Procedure Methods and Materno‐Fetal Complications ‐ A Literature Review,” Journal of Gynecology Obstetrics and Human Reproduction 51, no. 1 (2022): 102252, 10.1016/j.jogoh.2021.102252. 34638008

[14] M. H. Dave , K. Schmid , and M. Weiss , “Airway Dimensions From Fetal Life to Adolescence‐A Literature Overview,” Pediatric Pulmonology 53, no. 8 (2018): 1140–1146, 10.1002/ppul.24046. 29806162

[15] D. M. Mirsky , K. V. Shekdar , and L. T. Bilaniuk , “Fetal MRI: Head and Neck,” Magnetic Resonance Imaging Clinics 20, no. 3 (2012): 605–618, 10.1016/j.mric.2012.06.002. 22877957

[16] N. Kathary , D. I. Bulas , K. D. Newman , and R. L. Schonberg , “MRI Imaging of Fetal Neck Masses With Airway Compromise: Utility in Delivery Planning,” Pediatric Radiology 31, no. 10 (2001): 727–731, 10.1007/s002470100527. 11685443

[17] S. Saadi , N. Ben Abdeljelil , A. Ben Salem , F. Z. Chioukh , and N. Haj Salem , “Tracheal Agenesis Clinical Presentation in a Preterm Infant: Prenatal MRI Difficulties and Autopsy Findings,” Radiology Case Reports 15, no. 9 (2020): 1604–1608, 10.1016/j.radcr.2020.06.047. 32685079 PMC7355954

[18] H. Aoki , O. Miyazaki , S. Irahara , et al., “Value of Parametric Indexes to Identify Tracheal Atresia With or Without Fistula on Fetal Magnetic Resonance Imaging,” Pediatric Radiology 51, no. 11 (2021): 2027–2037, 10.1007/s00247-021-05092-x. 33988754

[19] D. A. Lazar , C. I. Cassady , O. O. Olutoye , et al., “Tracheoesophageal Displacement Index and Predictors of Airway Obstruction for Fetuses With Neck Masses,” Journal of Pediatric Surgery 47, no. 1 (2012): 46–50, 10.1016/j.jpedsurg.2011.10.022. 22244391

[20] T. W. Ng , Y. Xi , D. Schindel , et al., “Fetal Head and Neck Masses: MRI Prediction of Significant Morbidity,” American Journal of Roentgenology 212, no. 1 (2019): 215–221, 10.2214/ajr.18.19753. 30422714

[21] M. Szpinda , M. Daroszewski , A. Szpinda , et al., “New Quantitative Patterns of the Growing Trachea in Human Fetuses,” Medical Science Monitor 18, no. 6 (2012): PH63–PH70, 10.12659/msm.882890. 22648261 PMC3560714

[22] null Harjeet , D. Sahni , Y. K. Batra , and S. Rajeev , “Anatomical Dimensions of Trachea, Main Bronchi, Subcarinal and Bronchial Angles in Fetuses Measured Ex Vivo,” Paediatric Anaesthesia 18, no. 11 (2008): 1029–1034, 10.1111/j.1460-9592.2008.02775.x. 18950324

[23] M. Szpinda , M. Daroszewski , A. Woźniak , A. Szpinda , and C. Mila‐Kierzenkowska , “Tracheal Dimensions in Human Fetuses: An Anatomical, Digital and Statistical Study,” Surgical and Radiologic Anatomy 34, no. 4 (2012): 317–323, 10.1007/s00276-011-0878-7. 21984196 PMC3334485

[24] M. Daroszewski , M. Szpinda , P. Flisiński , et al., “Tracheo‐Bronchial Angles in the Human Fetus – An Anatomical, Digital, and Statistical Study,” Medical Science Monitor Basic Research 19 (2013): 194–200, 10.12659/msmbr.889085. 23857411 PMC3724572

[25] K. Harjeet , A. Aggarwal , D. Sahni , Y. K. Batra , S. V. Rakesh , and R. Subramanyam , “Anatomical Dimensions of Larynx, Epiglottis and Cricoid Cartilage in Foetuses and Their Relationship With Crown Rump Length,” Surgical and Radiologic Anatomy 32, no. 7 (2010): 675–681, 10.1007/s00276-010-0670-0. 20454794

[26] P. Fayoux , B. Marciniak , L. Devisme , and L. Storme , “Prenatal and Early Postnatal Morphogenesis and Growth of Human Laryngotracheal Structures,” Journal of Anatomy 213, no. 2 (2008): 86–92, 10.1111/j.1469-7580.2008.00935.x. 19172727 PMC2526109

[27] A. E. Cicekcibasi , B. Keles , and M. Uyar , “The Morphometric Development of the Fetal Larynx During the Fetal Period,” International Journal of Pediatric Otorhinolaryngology 72, no. 5 (2008): 683–691, 10.1016/j.ijporl.2008.02.002. 18359094

[28] D. Miklaszewska , A. Gawlikowska‐Sroka , F. Czerwiński , E. Dzieciołowska‐Baran , and E. Adamiec , “A Morphometric Study of Prenatal Development of the Human Larynx,” Annales Academiae Medicae Stetinensis 56, no. 3 (2010): 103–106, https://pubmed.ncbi.nlm.nih.gov/22053632/. 22053632

[29] K. D. Kalache , M. Franz , R. Chaoui , and R. Bollmann , “Ultrasound Measurements of the Diameter of the Fetal Trachea, larynx and Pharynx Throughout Gestation Applicability to Prenatal Diagnosis of Obstructive Anomalies of the Upper Respiratory‐Digestive Tract,” Prenatal Diagnosis 19, no. 3 (1999): 211–218, 10.1002/(sici)1097-0223(199903)19:3<211::aid-pd487>3.0.co;2-9. 10210118

[30] Morphological Features of Trachea in Developing Human Fetuses.

[31] T. Victoria , A. M. Johnson , J. C. Edgar , D. M. Zarnow , A. Vossough , and D. Jaramillo , “Comparison Between 1.5‐T and 3‐T MRI for Fetal Imaging: Is There an Advantage to Imaging With a Higher Field Strength?,” American Journal of Roentgenology 206, no. 1 (2016): 195–201, 10.2214/ajr.14.14205. 26700352

