# Supplementary material for: Prenatal Magnetic Resonance Imaging Assessment of Fetal Airway Dimensions: Establishing an Anthropometric Database
Source: Prenat Diagn. 2026 Apr 2;46(9):1330–8. doi: 10.1002/pd.70140 (PMC13446534; doi:10.1002/pd.70140)
Supplement: Supplementary file 1 [file PD-46-1330-s001.docx]

**Appendice 1.** Measured values by MRI by weeks of amenorrhea (mean (± standard deviation)). Tracheal diameters and lengths are expressed in 10^-3^ m, area in 10^-4^ m².

|  |  | Axial |  | Sagittal | | Coronal | Pharynx | |  |
| --- | --- | --- | --- | --- | --- | --- | --- | --- | --- |
| WA | N | Maximal diameter | Minimal diameter | Diameter | Length | Diameter | | Axial area | Sagittal area |
| 23 | 1 | 1.90 | 1.6 | 1.30 | 25.0 | 1.70 | | 0.52 | 1.34 |
| 25 | 1 | 2.20 | 1.5 | 2.20 | 21.9 | 2.10 | | 0.37 | 1.38 |
| 26 | 2 | 2.80 (±0.28) | 1.75 (±0.35) | 2.55 (±0.07) | 34.4 (±10.9) | 2.20 (±0.14) | | 0.62 (±0.09) | 1.47 (±0.25) |
| 27 | 6 | 2.45 (±0.36) | 1.67 (±0.31) | 2.00 (±0.64) | 30.5 (±12.5) | 2.17 (±0.14) | | 0.61 (±0.22) | 1.45 (±0.82) |
| 28 | 11 | 2.55 (±0.35) | 1.82 (±0.32) | 2.09 (±0.35) | 31.1 (±6.5) | 2.19 (±0.57) | | 0.57 (±0.18) | 0.97 (±0.28) |
| 29 | 12 | 2.34 (±0.42) | 1.58 (±0.29) | 1.98 (±0.33) | 26.5 (±6.8) | 2.46 (±0.45) | | 0.81 (±0.28) | 1.13 (±0.36) |
| 30 | 16 | 2.68 (±0.43) | 1.79 (±0.34) | 1.97 (±0.38) | 32.4 (±9.7) | 2.19 (±0.46) | | 0.69 (±0.30) | 1.15 (±0.39) |
| 31 | 25 | 2.69 (±0.40) | 1.98 (±0.43) | 2.35 (±0.51) | 34.6 (±8.8) | 2.58 (±0.52) | | 0.75 (±0.21) | 1.35 (±0.32) |
| 32 | 63 | 2.82 (±0.47) | 1.92 (±0.46) | 2.32 (±0.45) | 37.1 (±10.0) | 2.67 (±0.47) | | 0.93 (±0.43) | 1.48 (±0.54) |
| 33 | 47 | 3.08 (±0.61) | 1.99 (±0.43) | 2.27 (±0.47) | 36.8 (±9.1) | 2.84 (±0.62) | | 0.85 (±0.47) | 1.53 (±0.50) |
| 34 | 40 | 2.86 (±0.73) | 1.97 (±0.46) | 2.39 (±0.39) | 39.7 (±9.9) | 2.70 (±0.51) | | 0.96 (±0.51) | 1.47 (±0.49) |
| 35 | 26 | 2.97 (±0.68) | 2.03 (±0.42) | 2.50 (±0.70) | 36.3 (±7.2) | 2.89 (±0.65) | | 0.93 (±0.33) | 1.60 (±0.61) |
| 36 | 24 | 3.04 (±0.56) | 2.24 (±0.50) | 2.26 (±0.44) | 38.9 (±8.9) | 3.18 (±0.53) | | 0.95 (±0.36) | 1.29 (±0.42) |
| 37 | 11 | 3.06 (±0.67) | 1.98 (±0.52) | 2.44 (±0.57) | 36.5 (±11.9) | 2.91 (±0.77) | | 0.93 (±0.38) | 1.54 (±0.65) |
| 38 | 8 | 3.20 (±0.72) | 2.11 (±0.39) | 2.75 (±0.61) | 43.4 (±12.2) | 3.24 (±0.69) | | 0.93 (±0.52) | 1.72 (±0.91) |
| 39 | 2 | 2.10 (±0.00) | 1.85 (±0.35) | 2.10 (±0.42) | 33.3 (±18.6) | 2.45 (±0.21) | | 0.52 | 1.57 (±0.54) |
| 41 | 1 | 3.00 | 1.90 | 2.20 | 44.9 | 3.50 | | 0.28 | 1.09 |

**Appendice 2.** Measured values by MRI values by sex (mean (± standard deviation)). Trachea diameters and lengths in 10^-3^ m, area in 10^-4^ m^2^, volume in 10^-6^ m^3^ and angles in degrees.

|  | Females  (n = 140) | | | Males  (n = 155) | | | | p value | |
| --- | --- | --- | --- | --- | --- | --- | --- | --- | --- |
| Trachea |  |  | |  | |  | |  | |
| Axial |  |  | |  | |  | |  | |
| Minimal Diameter | 1.92 (±0.43) | | | | 1.98 (±0.46) | | | | 0.279 |
| Maximal Diameter | 2.80 (±0.59) | | | | 2.90 (±0.59) | | | | 0.133 |
| Sagittal |  | | | |  | | | |  |
| Diameter | 2.23 (±0.48) | | | | 2.37 (±0.50) | | | | 0.017* |
| Length | 35.4 (±9.6) | | | | 37.1 (±9.9) | | | | 0.134 |
| Coronal |  | | | |  | | | |  |
| Diameter | 2.67 (±0.59) | | | | 2.76 (±0.60) | | | | 0.191 |
| Subcarinal angle |  | | | |  | | | |  |
| <30 | 4 (2.9%) | | | | 9 (5.8%) | | | | 0.171 |
| 30-60 | 73 (52.5%) | | | | 69 (44.5%) | | | |  |
| 60-90 | 50 (36.0%) | | | | 69 (44.5%) | | | |  |
| >90 | 12 (8.6%) | | | | 8 (5.2%) | | | |  |
| Left angle |  | | | |  | | | |  |
| <90 | 0 (0.0%) | | | | 4 (2.6%) | | | | 0.385 |
| 90-120 | 11 (7.9%) | | | | 12 (7.7%) | | | |  |
| 120-150 | 93 (66.4%) | | | | 94 (60.6%) | | | |  |
| 150-180 | 35 (25.0%) | | | | 43 (27.7%) | | | |  |
| >180 | 1 (0.7%) | | | | 2 (1.3%) | | | |  |
| Right angle |  | | | |  | | | |  |
| <120 | 2 (1.4%) | | | | 9 (5.8%) | | | | 0.247 |
| 120-140 | 14 (10.0%) | | | | 17 (11.0%) | | | |  |
| 140-160 | 60 (42.9%) | | | | 59 (38.1%) | | | |  |
| 160-180 | 61 (43.6%) | | | | 69 (44.5%) | | | |  |
| >180 | 3 (2.1%) | | | | 1 (0.6%) | | | |  |
| **Other measurements** |  | |  | |  | |  | |  |
| Axial pharyngeal area | 0.86 (±0.42) | | | | 0.85 (±0.39) | | | | 0.898 |
| Sagittal pharyngeal area | 1.42 (±0.49) | | | | 1.44 (±0.55) | | | | 0.773 |

**Appendice 3.** Measured values by MRI according to fetal presentation (mean (± standard deviation)). Trachea diameters and lengths in 10^-3^ m, volume in 10^-6^ m^3^ and angles in degrees.

|  | | Cephalic  (n = 95) | | Back right  (n = 53) | | | Back left  (n = 84) | | | | Posterior back  (n = 8) | | Anterior back  (n = 14) | | | | p value | |
| --- | --- | --- | --- | --- | --- | --- | --- | --- | --- | --- | --- | --- | --- | --- | --- | --- | --- | --- |
| Trachea |  | | | |  | | |  | |  | | | |  | |  | | |
| Axial |  | | | |  | | |  | |  | | | |  | |  | | |
| Minimal Diameter | 1.93 (±0.43) | | | | 2.05 (±0.40) | | | 1.97 (±0.48) | | 2.06 (±0.32) | | | | 1.77 (±0.50) | | 0.140 | | |
| Maximal Diameter | 2.86 (±0.53) | | | | 2.98 (±0.51) | | | 2.85 (±0.70) | | 2.98 (±0.41) | | | | 2.83 (±0.56) | | 0.518 | | |
| Sagittal |  | | | |  | | |  | |  | | | |  | |  | | |
| Diameter | 2.35 (±0.53) | | | | 2.21 (±0.44) | | | 2.32 (±0.49) | | 2.51 (±0.47) | | | | 2.23 (±0.43) | | 0.359 | | |
| Length | 35.1 (±10.4) | | | | 37.4 (±9.6) | | | 37.7 (±9.2) | | 37.7 (±11.6) | | | | 36.2 (±10.9) | | 0.516 | | |
| Coronal |  | | | |  | | |  | |  | | | |  | |  | | |
| Diameter | 2.72 (±0.63) | | | | 2.67 (±0.54) | | | 2.75 (±0.65) | | 2.85 (±0.52) | | | | 2.66 (±0.60) | | 0.935 | | |
| Subcarinal angle | | |  | | |  | | |  | | |  | | |  | | |  |
| <30 | 8 (8.5%) | | | | 2 (3.8%) | | | 0 (0.0%) | | 1 (12.5%) | | | | 1 (7.1%) | |  | | |
| 30-60 | 41 (43.6%) | | | | 28 (52.8%) | | | 46 (54.8%) | | 3 (37.5%) | | | | 7 (50.0%) | |  | | |
| 60-90 | 38 (40.4%) | | | | 18 (34.0%) | | | 34 (40.5%) | | 3 (37.5%) | | | | 5 (35.7%) | |  | | |
| >90 | 7 (7.4%) | | | | 5 (9.4%) | | | 4 (4.8%) | | 1 (12.5%) | | | | 1 (7.1%) | |  | | |
| Left angle |  | | | |  | | |  | |  | | | |  | |  | | |
| <90 | 2 (2.1%) | | | | 0 (0.0%) | | | 0 (0.0%) | | 0 (0.0%) | | | | 1 (7.1%) | |  | | |
| 90-120 | 3 (3.2%) | | | | 2 (3.8%) | | | 14 (16.7%) | | 2 (25.0%) | | | | 1 (7.1%) | |  | | |
| 120-150 | 62 (65.3%) | | | | 28 (52.8%) | | | 54 (64.3%) | | 2 (25.0%) | | | | 8 (57.1%) | |  | | |
| 150-180 | 27 (28.4%) | | | | 22 (41.5%) | | | 15 (17.9%) | | 4 (50.0%) | | | | 4 (28.6%) | |  | | |
| >180 | 1 (1.1%) | | | | 1 (1.9%) | | | 1 (1.2%) | | 0 (0.0%) | | | | 0 (0.0%) | |  | | |
| Right angle |  | | | |  | | |  | |  | | | |  | |  | | |
| <120 | 2 (2.1%) | | | | 3 (5.7%) | | | 4 (4.8%) | | 0 (0.0%) | | | | 1 (7.1%) | |  | | |
| 120-140 | 12 (12.6%) | | | | 8 (15.1%) | | | 3 (3.6%) | | 2 (25.0%) | | | | 1 (7.1%) | |  | | |
| 140-160 | 43 (45.3%) | | | | 20 (37.7%) | | | 28 (33.3%) | | 1 (12.5%) | | | | 5 (35.7%) | |  | | |
| 160-180 | 36 (37.9%) | | | | 22 (41.5%) | | | 48 (57.1%) | | 4 (50.0%) | | | | 7 (50.0%) | |  | | |
| >180 | 2 (2.1%) | | | | 0 (0.0%) | | | 1 (1.2%) | | 1 (12.5%) | | | | (0.0%) | |  | | |

**Appendice 4.** Measured values by MRI according to fetal presentation (mean (± standard deviation)).. Areas are expressed in 10^-4^ m^2^.

|  | Cephalic  (n = 95) | | Back right  (n = 53) | | | Back left  (n = 84) | | | Posterior back  (n = 8) | | | Anterior back  (n = 14) | | | p value | | |  |
| --- | --- | --- | --- | --- | --- | --- | --- | --- | --- | --- | --- | --- | --- | --- | --- | --- | --- | --- |
| Axial pharyngeal area | 0.83 (±0.43) | | | 0.98 (±0.42) | | | 0.81 (±0.36) | | | 0.93 (±0.40) | | | 0.81 (±0.22) | | | 0.252 | | |
| Sagittal pharyngeal area | 1.39 (±0.56) | | | 1.56 (±0.51) | | | 1.50 (±0.50) | | | 1.38 (±0.68) | | | 1.35 (±0.60) | | | 0.104 | | |

**Appendice 5.** Measured values by MRI according to sagittal tilt of the head (mean (± standard deviation)).. Areas are expressed in 10^-4^ m^2^. “Head flexed” corresponds to a head flexion angle of less than 60°, “in axis” corresponds to a head flexion angle of between 60° and 120°, and “hyperextension” corresponds to an angle greater than 120°.

|  | Head flexed  (n = 130) | In axis  (n = 103) | Hyperextension  (n = 9) | p value |
| --- | --- | --- | --- | --- |
| Axial pharyngeal area | 0.91 (±0.41) | 0.73 (±0.31) | 0.89 (±0.30) | 0.018* |
| Sagittal pharyngeal area | 1.36 (±0.45) | 1.54 (±0.56) | 1.66 (±0.65) | 0.031* |

**Appendice 6.** Measured values by MRI according to sagittal head tilt (mean (± standard deviation)).. Trachea diameters and lengths in 10^-3^ m, volume in 10^-6^ m^3^, and angles in degrees. “Head flexed” corresponds to a head flexion angle of less than 60°, “in axis” corresponds to a head flexion angle of between 60° and 120°, and “hyperextension” corresponds to an angle greater than 120°.

|  | Head flexed  (n = 130) | | | In axis  (n = 103) | | | | Hyperextension  (n = 9) | | | | | p value | | | | |
| --- | --- | --- | --- | --- | --- | --- | --- | --- | --- | --- | --- | --- | --- | --- | --- | --- | --- |
| Trachea |  | |  |  | | | |  | | | |  |  | | | | |
| Axial | |  | | | | |  | | | |  | | | | |  | |
| Minimal Diameter | | 1.98 (±0.49) | | | | | 1.99 (±0.41) | | | | 1.64 (±0.30) | | | | | 0.057 | |
| Maximal Diameter | | 2.93 (±0.59) | | | | | 2.84 (±0.59) | | | | 2.58 (±0.55) | | | | | 0.175 | |
| Sagittal | |  | | | | |  | | | |  | | | | |  | |
| Diameter | | 2.31 (±0.47) | | | | | 2.26 (±0.52) | | | | 2.20 (±0.63) | | | | | 0.469 | |
| Length | | 35.8 (±9.4) | | | | | 35.9 (±9.4) | | | | 44.9 (±12.4) | | | | | 0.034* | |
| Coronal | |  | | | | |  | | | |  | | | | |  | |
| Diameter | | 2.75 (±0.58) | | | | | 2.69 (±0.63) | | | | 2.71 (±0.62) | | | | | 0.512 | |
| Subcarinal angle | | | | | |  | | | |  | | | | |  | | |
| <30 | 5 (3.9%) | | | | 5 (4.9%) | | | | 0 (0.0%) | | | | | 0.943 | | |  |
| 30-60 | 64 (49.6%) | | | | 52 (50.5%) | | | | 4 (44.4%) | | | | |  | | |  |
| 60-90 | 54 (41.9%) | | | | 39 (37.9%) | | | | 5 (55.6%) | | | | |  | | |  |
| >90 | 6 (4.7%) | | | | 7 (6.8%) | | | | 0 (0.0%) | | | | |  | | |  |
| Left angle |  | | | |  | | | |  | | | | |  | | |  |
| <90 | 1 (0.8%) | | | | 1 (1.0%) | | | | 0 (0.0%) | | | | | 0.552 | | |  |
| 90-120 | 9 (6.9%) | | | | 7 (6.8%) | | | | 2 (22.2%) | | | | |  | | |  |
| 120-150 | 86 (66.2%) | | | | 61 (59.2%) | | | | 6 (66.7%) | | | | |  | | |  |
| 150-180 | 32 (24.6%) | | | | 33 (32.0%) | | | | 1 (11.1%) | | | | |  | | |  |
| >180 | 2 (1.5%) | | | | 1 (1.0%) | | | | 0 (0.0%) | | | | |  | | |  |
| Right angle |  | | | |  | | | |  | | | | |  | | |  |
| <120 | 5 (3.8%) | | | | 2 (1.9%) | | | | 0 (0.0%) | | | | | 0.818 | | |  |
| 120-140 | 11 (8.5%) | | | | 12 (11.7%) | | | | 1 (11.1%) | | | | |  | | |  |
| 140-160 | 59 (45.4%) | | | | 44 (42.7%) | | | | 3 (33.3%) | | | | |  | | |  |
| 160-180 | 54 (41.5%) | | | | 42 (40.8%) | | | | 5 (55.6%) | | | | |  | | |  |
| >180 | 1 (0.8%) | | | | 3 (2.9%) | | | | 0 (0.0%) | | | | |  | | |  |

**Appendice 7.** Measured values by MRI according to lateral head tilt (mean (± standard deviation)).. Trachea diameters and lengths in 10^-3^ m, volume in 10^-6^ m^3^, and angles in degrees. “Head tilt” corresponds to an angle of lateral inclination less than 112.5° or greater than 157.5°, “on axis” corresponds to an angle between 112.5° and 157.5°.

|  | | Head tilt  (n = 125) | | On axis  (n = 137) | | | p value | |
| --- | --- | --- | --- | --- | --- | --- | --- | --- |
| Trachea | |  |  |  | |  |  | |
| Axial |  | | | |  | | |  |
| Minimal Diameter | 1.98 (±0.44) | | | | 1.92 (±0.44) | | | 0.276 |
| Maximal Diameter | 2.90 (±0.54) | | | | 2.81 (±0.61) | | | 0.245 |
| Sagittal |  | | | |  | | |  |
| Diameter | 2.27 (±0.50) | | | | 2.31 (±0.50) | | | 0.510 |
| Length | 34.6 (±9.6) | | | | 36.6 (±9.7) | | | 0.095 |
| Coronal |  | | | |  | | |  |
| Diameter | 2.72 (±0.60) | | | | 2.72 (±0.60) | | | 0.790 |
| Subcarinal angle |  | | | |  | | |  |
| <30 | 6 (4.8%) | | | | 4 (2.9%) | | | 0.140 |
| 30-60 | 60 (48.4%) | | | | 71 (51.8%) | | |  |
| 60-90 | 55 (44.4%) | | | | 51 (37.2%) | | |  |
| >90 | 3 (2.4%) | | | | 11 (8.0%) | | |  |
| Left angle |  | | | |  | | |  |
| <90 | 0 (0.0%) | | | | 3 (2.2%) | | | 0.378 |
| 90-120 | 11 (8.8%) | | | | 8 (5.8%) | | |  |
| 120-150 | 78 (62.4%) | | | | 92 (67.2%) | | |  |
| 150-180 | 35 (28.0%) | | | | 32 (23.4%) | | |  |
| >180 | 1 (0.8%) | | | | 2 (1.5%) | | |  |
| Right angle |  | | | |  | | |  |
| <120 | 5 (4.0%) | | | | 3 (2.2%) | | | 0.004* |
| 120-140 | 5 (4.0%) | | | | 21 (15.3%) | | |  |
| 140-160 | 57 (45.6%) | | | | 44 (32.1%) | | |  |
| 160-180 | 55 (44.0%) | | | | 68 (49.6%) | | |  |
| >180 | 3 (2.4%) | | | | 1 (0.7%) | | |  |

**Appendice 8.** Measured values by MRI according to lateral head tilt (mean (± standard deviation)).. Areas are expressed in 10^-4^ m^2^. “Head tilt” corresponds to an angle of lateral inclination of less than 112.5° or greater than 157.5°, “on axis” corresponds to an angle between 112.5° and 157.5°.

|  | Head tilt  (n = 125) | On axis  (n = 137) | p value |
| --- | --- | --- | --- |
| Axial pharyngeal area | 0.80 (±0.36) | 0.88 (±0.40) | 0.183 |
| Sagittal pharyngeal area | 1.40 (±55.1) | 1.48 (±50.6) | 0.151 |
